# Self-Floating Polydopamine/Polystyrene Composite Porous Structure via a NaCl Template Method for Solar-Driven Interfacial Water Evaporation

**DOI:** 10.3390/polym16152231

**Published:** 2024-08-05

**Authors:** Xiao Wang, Zhen Li, Xiaojing Wu, Bingjie Liu, Tian Tian, Yi Ding, Haibo Zhang, Yuanli Li, Ye Liu, Chunai Dai

**Affiliations:** 1 School of Undergraduate Education, Shenzhen Polytechnic University, Shenzhen 518055, China; 23126690@bjtu.edu.cn; 2 School of Physical Science and Engineering, Beijing Jiaotong University, Beijing 100044, China; ttian@bjtu.edu.cn (T.T.); 23121824@bjtu.edu.cn (Y.D.); 22341149@bjtu.edu.cn (H.Z.); 23342012@bjtu.edu.cn (Y.L.); 22341062@bjtu.edu.cn (Y.L.); 3 Advanced Materials and Energy Center, Academy of Aerospace Science and Innovation, Beijing 100088, China; chemlizhen@gmail.com; 4 Beijing Institute of Space Mechanics & Electricity, Beijing 100094, China; bingjie1118@outlook.com

**Keywords:** polydopamine, polystyrene, photothermal materials, porous structure, NaCl template method, solar-driven interfacial water evaporation

## Abstract

Solar energy, as a clean and renewable energy source, holds significant promise for addressing water shortages. Utilizing solar energy for water evaporation is seen as an effective solution in this regard. While many existing interfacial photothermal water evaporation systems rely on nanoparticles or graphene as photothermal or support materials, this study introduced polydopamine (PDA) as a photothermal material due to its environmental friendliness and excellent photon absorption characteristics that closely match the solar spectrum. Polystyrene (PS) was also introduced as a support material for its porous structure and density similar to water, enabling it to float on water. The resulting PS-PDA composite porous structure solar evaporator exhibited a photothermal conversion efficiency comparable to nanoparticles (over 75%), yet with lower production costs and minimal environmental impact. This innovative approach offers a scalable solution for water-scarce regions, providing a cost-effective and efficient means to address water scarcity. The use of PDA and PS in this context highlights the potential for utilizing common materials in novel ways to meet pressing environmental challenges.

## 1. Introduction

The global water shortage issue is rapidly escalating, impacting approximately one-third of the world’s population. This crisis has emerged as one of the most pressing challenges of the 21st century. The lack of access to safe and clean water not only affects human health but also poses significant challenges to agriculture, industry, and ecosystems [1,2,3,4]. Solar-driven water evaporation—the extraction of vapor from liquid water using solar energy—provides the basis for the development of freshwater production. Traditional solar water evaporation systems use their surfaces or cavities to absorb solar radiation and then transfer heat to the water. This method produces a large amount of energy loss due to the influence of thermal radiation and convection, and the photothermal conversion efficiency is not high.

To this end, an interfacial evaporation system floating on the water was proposed, which can lock thermal energy at the interface to minimize heat loss and improve conversion efficiency [5,6]. The materials that make up the interfacial evaporation system should have excellent photothermal conversion properties and good water transmission properties. In recent years, interfacial evaporation materials with different structural designs and excellent properties have been proposed, including porous graphene oxide [7,8], gold, aluminum, copper, silicon, and nanoparticles such as manganese dioxide, titanium dioxide, molybdenum disulfide, iron oxide, cobalt oxide, copper sulfide, and so on [9,10,11,12,13,14,15,16,17,18,19]. However, these are non-biodegradable photothermal materials and support materials. Once performance degradations occur, they will not only affect the photothermal conversion efficiency but also have certain impacts on the environment. The extent of the impact of these materials currently used in large quantities on interface evaporator systems entering the environment remains a matter of uncertainty and requires further discussion [20,21]. On this basis, the choice of interface evaporator material must take into account not only the photothermal conversion efficiency and water transport properties but also the environmental impact of the material itself.

Polydopamine (PDA) is a black biopolymer formed by the oxidative self-polymerization of dopamine (DA) and can be used as a coating to adhere to a variety of materials and as a platform for surface reactions [22]. PDA has good photothermal properties and can absorb the vast majority of photon energy in the solar spectrum and convert it into heat [23,24]. Moreover, PDA has good biodegradability [25] and PDA is harmless in mammalian cells [26,27,28]. Therefore, PDA can be used as a multifunctional surface treatment material with huge application prospects in biology, energy, and industry [29,30,31].Polystyrene (PS) is a widely used thermoplastic polymer known for its cost-effectiveness, stable physical and chemical properties, and excellent biocompatibility [32,33]. Its recyclability further adds to its appeal, making it a primary material in everyday packaging, disposable containers, and insulation products [34,35]. One of its key advantages is its compatibility with PDA, making it an optimal support material for PDA loading [36,37,38,39]. Its compatibility and versatility make PS a compelling choice as a support material in various studies.

In this study, we investigated the composite formation of PS with PDA at varying concentrations. Based on the composite strategy, we developed a novel PS-PDA composite porous structure solar-driven interfacial water evaporator. The design exhibited outstanding water vapor collection efficiency under specific solar irradiation intensities. Additionally, the PS-PDA composite porous structure solar evaporator demonstrated low raw material costs and a simple, environmentally friendly preparation process, which shows significant potential for large-scale production and utilization. The characteristics of low cost and high-water vapor collection efficiency make the PS-PDA evaporator a promising solution for water-scarce regions. This advancement not only provides a sustainable method for water evaporation but also addresses the global challenge of water scarcity in a practical and scalable manner.

## 2. Materials and Methods

### 2.1. Materials and Characterization

In this study, dopamine hydrochloride (DA·HCl, 98%, TCI Chemicals, Shanghai, China), PS (98%, Aladdin’s reagent, Shanghai, China), sodium chloride (NaCl, AR, Aladdin’s reagent, Shanghai, China), toluene (AR, Aladdin’s reagent, Shanghai, China), ethanol (AR, Beijing Chemical Factory, Beijing, China) and ammonia (28%, AR, Beijing Chemical Plant, Beijing, China) were used. All materials were used directly without processing.

A scanning electron microscope (SEM, Hitachi SU8000, Tokyo, Japan) was employed to photograph the samples. Absorbance or reflectance of PS-PDA was tested using UV–VIS–NIR diffuse reflection spectroscopy (DRS, Hitachi, U-3010, Tokyo, Japan). The water contact angle was measured using an OCA 15Pro instrument (Dataphysics, Stuttgart, Germany), and an infrared thermal imager (FLIR E40, Wilsonville, OR, USA) was used for measuring the temperature of the object.

### 2.2. Synthesis of PDA Nanoparticles

PDA nanoparticles were synthesized following previous literature reports [27,40,41]. The specific synthesis method is as follows.

A mixed solution of 180 mL deionized water and 80 mL ethanol was prepared, to which 5 mL of 28–30% ammonia water was added, and the mixed solution was stirred at room temperature for 10 min.

Subsequently, 1 g of DA·HCl was dissolved in 20 mL of deionized water and added dropwise to the mixed solution above; after stirring for 30 min, the mixture was then left at room temperature for 24 h to allow for the polymerization of DA.

The PDA nanoparticles were obtained by centrifuging the reaction mixture at 8000 rpm multiple times, washing the precipitate with water, and freeze-drying. 

### 2.3. Fabrication of PS-PDA Material

**Figure 1:**
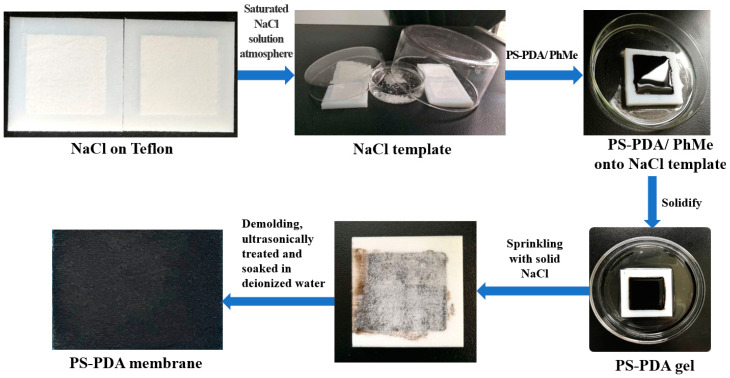
Schematic diagram of the preparation of PS-PDA composite porous structure material.

The production of the PS-PDA composite porous structure solar evaporator for photoevaporation of water was modified based on the literature [42]. It was divided into three steps as shown in Figure 1. The first step was to make a toluene solution of PS-PDA(PS-PDA/PhMe), which was prepared for later use by placing 1.32 g of PS, 4 mL of toluene, and 1, 3, 5, or 7% PDA nanoparticles in a small glass bottle and stirring for 24 h. The reason for choosing toluene is that it is a common organic solvent in the laboratory and has good solubility and low toxicity. The second step was to fabricate a PS-PDA gel. First, 200-mesh NaCl was ground to a Teflon template with a depth of 0.5 mm and placed in an atmosphere of saturated NaCl solution to solidify. The above PS-PDA/PhMe solution with different PDA concentrations was then dropwise added to the hardened NaCl template and placed in a toluene atmosphere for percolation until no bubbles escaped. The third step was curing and demolding. After scraping off the liquid on the surface with a scraper, the PS-PDA gel on the NaCl template was sprinkled with NaCl particles and put in a fume hood to solidify in the air. This step aims to create more porous channels in the surface. The cured PS-PDA was then de-molded, ultrasonically treated, and soaked in deionized water for 24 h to remove NaCl. For PS-PDA materials prepared with this template method, the surface formed on the air interface side is named the top and the surface formed on the Teflon interface side is named the bottom. The PS template followed the same preparation method, except that the PDA was not added.

### 2.4. Solar-Driven Interfacial Water Evaporation Measurements

Solar-driven interfacial water evaporation measurements were conducted indoors and outdoors. In the case of indoors, a solar simulator composed of a xenon light source (Beijing Zhongjiao Jinyuan Technology Corporation CEL-S500, Beijing, China) was used, surrounded by thermal insulation glass to avoid heat loss. A 3 cm × 3 cm × 10 cm sample stage was placed in the center on which the solar water evaporator was placed. The weight of the lost water was measured by a precision balance (Mettler Toledo ME204, Zurich, Switzerland) placed under the sample stage. Before testing, a solar standard cell (Oriel Newport/VLSI 91150V, Newport, RI, USA) was used for solar intensity calibration. While testing, the solar water evaporator of the PS-PDA material was placed and floated on the water in a quartz cup. The water evaporated from the interface of the solar evaporator due to the heat generated by the xenon. When the evaporation time reached 15 min, it was considered to be balanced. In the case of outdoors, the measurements were carried out in sunlight. Our outdoor experiments took place in Beijing during the summer, with an average maximum weather temperature of 36 °C throughout the week. We conducted the tests in the afternoon when the sun was at its strongest.

## 3. Results

### 3.1. Morphology of PS-PDA Materials

**Figure 2:**
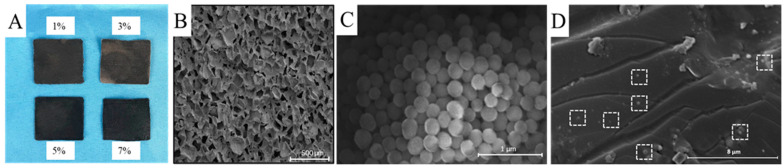
(**A**) The actual photos of the PS-PDA composite porous structure materials with different PDA contents. SEM images of (**B**) PS, (**C**) PDA, and (**D**) PS-PDA with 5% PDA doped.

Figure 2 shows the photos and SEM images of the prepared PS-PDA materials. The actual photos of the PS-PDA composite porous structure materials with different PDA contents are shown in Figure 2A. As the PDA content increases, the color of the PS-PDA material gradually deepens. Figure 2B is an SEM image of a PS support material. It can be seen from the picture that the PS has a highly open porous structure. The average aperture of the PS measured using the Nano Measurer software (V1.2.0) is 76 μm. Figure 2C shows an SEM image of the PDA particles. The PDA particles are spherical, with an average diameter of approximately 300 nm measured using Nano Measurer software. Figure 2D shows the SEM image of the PS-PDA with 5% PDA doped. It can be observed that the PDA particles (dotted) were loaded into the PS support material. Based on these morphology studies, it can be estimated that highly dense PDA particles can be accommodated in the pores of PS, and the porous nature of PS also facilitates the transmission of water from the liquid to the evaporation surface, greatly improving the water transmission efficiency.

### 3.2. Wettability of PS-PDA Materials

**Figure 3:**
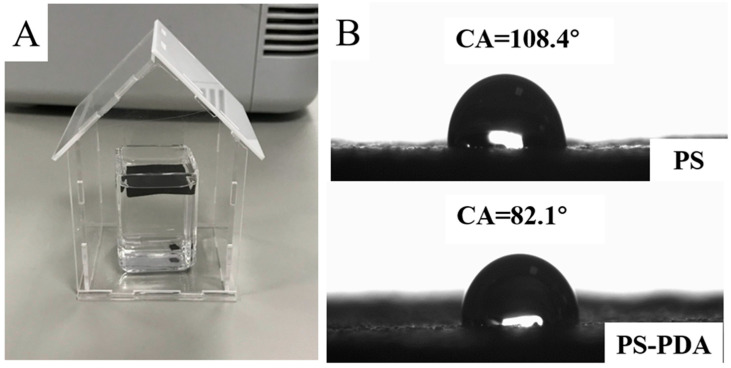
(**A**) Picture of the PS-PDA composite evaporator floating on the water. (**B**) Water contact angle test: the picture above shows the water droplet (4 μL) on PS and the picture below shows the water droplet (4 μL) on PS-PDA.

We tested the wettability of the PS-PDA material with 5% PDA doped; the results are listed in Figure 3. As shown in Figure 3A, the evaporator can self-float well at the air–water interface. One of the reasons for choosing PS as the support material is that the density of PS is close to that of water. This property will help the evaporator float on the water, maximizing the surface exposed to sunlight. Figure 3B shows the water contact angle test results of PS and PS-PDA materials. The water contact angle of 108° indicates that PS is a hydrophobic material. The picture below shows that PS loaded with 5% PDA particles has a decreased water contact angle of 82°, indicating that the surface of PS-PDA is more hydrophilic. Because the loaded PDA particles have exposed hydrophilic polar groups such as amines and hydroxyl groups [43], which increases the number of hydrophilic sites on the PS-PDA surface and improves the surface wettability, the PS-PDA evaporator has a stronger ability to transport water to the heat-absorbing surface. Moreover, the enhanced surface hydrophilicity of the PS-PDA evaporator can promote the adsorption of pollutants in water, indicating its potential as a material for water pollutant treatment. These findings highlight the multifaceted capabilities and promising applications of the PS-PDA composite material in water treatment and solar evaporation systems. The composite’s ability to adsorb pollutants, coupled with its potential for use in solar evaporation systems, underscores its versatility and potential impact in addressing water treatment challenges [12].

### 3.3. Light Absorption Property of PS-PDA Materials

**Figure 4:**
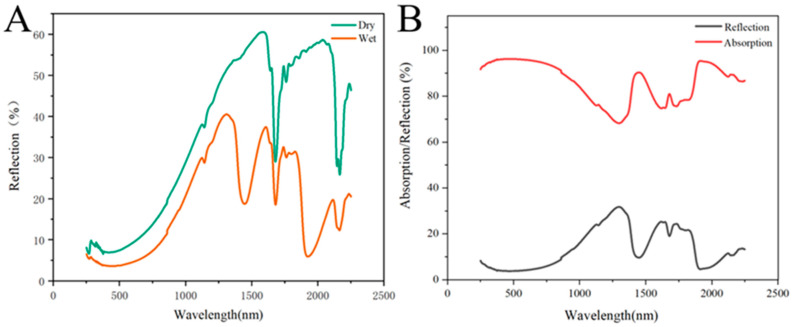
(**A**) UV–VIS–NIR DRS spectra of dry and wet PS-PDA membranes. (**B**) UV–VIS–NIR DRS spectra of wet PS-PDA membrane.

The UV–VIS–NIR DRS spectra of PS-PDA material with 5% PDA doped are shown in Figure 4. Figure 4A shows the spectra of the dry and wet PS-PDA materials. The reflectivity of the wet PS-PDA significantly decreased, indicating that the material had better solar light absorption performance in water. As shown in Figure 4B, the wet PS-PDA had a relatively low reflectivity (R); since the PS-PDA material had almost no transmitted light (T), the absorption rate A can be calculated using the formula A = 1 – T − R [44]. The calculated average absorption value over the entire solar spectral range reached 90%, indicating that the wet PS-PDA material has excellent solar light absorption performance and is therefore a candidate material for solar water evaporation applications.

### 3.4. Water Evaporation Test under Simulated Solar Radiation Indoors

**Figure 5:**
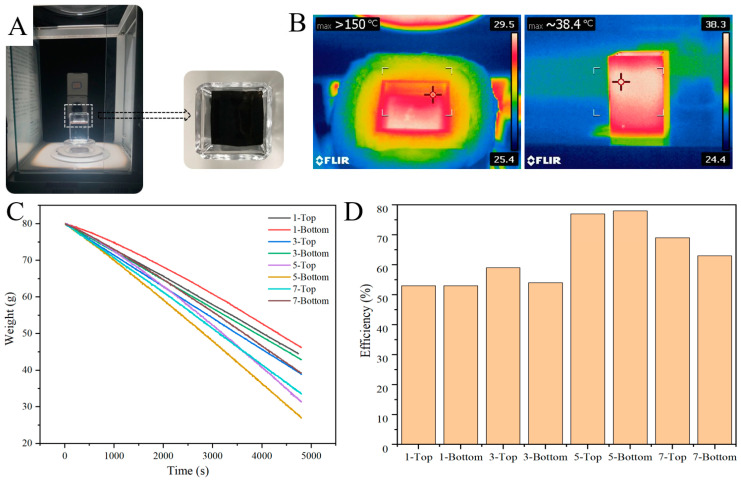
Water evaporation test under simulated solar radiation indoors. (**A**) Physical diagram of the photothermal conversion water vapor collection test device. (**B**) Infrared imaging of the photothermal conversion water vapor collection test device, where the left is for the PS-PDA material and the right is for the water. (**C**) Photothermal conversion water vapor collection test results of PS-PDA evaporators with different proportions of PDA doped. (**D**) Water vapor collection efficiency of PS-PDA with different proportions of PDA doped.

Figure 5A shows the photothermal conversion water vapor collection test device, in which the enlarged part is a top-view photo. The device was surrounded by insulated glass and equipped with a sample stage that could measure weight. This device simulated sunlight evaporating water and reflected the quality of the evaporation by measuring the amount of water reduced in the cup. Figure 5B represents the infrared imaging images of the photothermal conversion water vapor collection test device after 15 min of simulated solar irradiation, The cross head represents the highest temperature position in the diagram. The left image shows the temperature of the PS-PDA material and the maximum temperature has exceeded 150 °C. The right image depicts the temperature of the test water, with the highest recorded temperature being approximately 38.4 °C. The infrared imaging images indicate that the heat was focused within the material rather than in the water. This is due to the good photothermal properties of the PDA, which absorbs most of the photons of the solar spectrum and converts them into thermal energy [23,24]. During the photothermal conversion water vapor collection test, PS-PDA materials containing varying amounts of PDA were tested with their top and bottom surfaces facing the simulated sun to avoid uneven distribution of PDA. The results are shown in Figure 5C. For PS-PDA doped with 1% to 7% PDA, as the doped PDA concentration increased, the water evaporation rate first increased and then decreased. The evaporation rate peaked at 5% PDA doping. Because PDA has favorable photo-thermal characteristics, the water evaporation rate increases with the increase in PDA content. However, once the PDA content exceeds a certain level, the PDA will absorb too much light and heat, thus preventing the heat from reaching the water layer below. As a result, the water layer lacks the necessary heat from the light source to reach the required temperature for evaporation, leading to a decrease in the evaporation. 

The water vapor collection efficiency can be calculated by the following commonly used formula in this field [6,44]:

(1) $$\eta =\frac{mH_{LV}}{E_{i}}$$

In this equation, *m* is the mass of water that decreases per unit time and unit area, *H_LV_* is the evaporation enthalpy, and *E_i_* is the light intensity.

According to the given formula, the water vapor collection efficiency of PS-PDA with PDA content ranging from 1% to 7% has been calculated and is illustrated in Table 1 and Figure 5D. Remarkably, for PS-PDA with 5% PDA doping, the efficiency under one sun has exceeded 75%, higher than that of metal nanoparticles and graphene oxide. These efficiencies highlight the competitive performance of PS-PDA photothermal materials, showcasing the potential of PDA-PDA materials in solar-driven applications for water treatment and evaporation systems. This is because PDA has an absorption spectrum that closely matches the solar spectrum, and the porous nature of PS-PDA helps water transport from the liquid to the surface for evaporation.

### 3.5. Solar-Driven Interfacial Water Evaporation Test Outdoors

**Figure 6:**
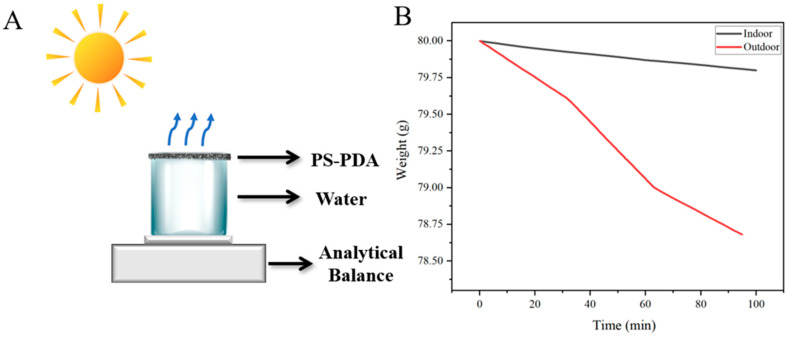
Outdoor test of water vapor collection. (**A**) Schematic diagram of the outdoor photothermal conversion water vapor collection test device. (**B**) Comparison of indoor and outdoor water vapor collection.

To explore the practical application possibilities of the PS-PDA evaporators, we further performed water vapor collection tests on the 5% PDA-doped PS-PDA interface evaporator under outdoor solar radiation, as shown in Figure 6A. For comparison, contrast tests were conducted under indoor conditions of 25 °C and 52% air humidity, as well as outdoor conditions of 36 °C and 56% air humidity. The results are plotted in Figure 6B. During the indoor test, the mass of the test water decreased from 80 g to 79.76 g. The relatively weak indoor sunlight led to a lower photon energy absorption by the PS-PDA interface evaporator. In contrast, during the outdoor testing, the mass of the test water decreased from 79.8 g to 78.44 g. The efficiency observed in the outdoor test is in close agreement with the results of other photothermal conversion water vapor harvesting devices. These findings support the practical applicability of the PS-PDA evaporators in real-world settings.

## 4. Conclusions

In this study, we present a novel solar evaporator design with exceptional performance metrics, including high photothermal conversion efficiency, cost-effectiveness, and environment-friendly. The solar evaporator is a PS-PDA composite porous structure prepared through a NaCl template method. The absorption spectrum of PDA particles closely matches the photon absorption characteristics of the solar spectrum, while PS’s porous structure and density that are similar to water enable its self-floating manner. This composite porous structure exhibits not only high photothermal conversion efficiency but also efficient water transmission. Our study demonstrates that a 5% PDA doping level in PS achieves the optimal water vapor collection efficiency, surpassing 75%. One of the key advantages of this solar evaporator is its use of readily available and environmentally friendly materials. The raw materials, including food-grade PS and biomass-derived PDA, are common and inexpensive. Additionally, the preparation process is simple and scalable, making it suitable for large-scale applications. Overall, the PS-PDA solar evaporator represents a significant advancement in solar evaporation technology, offering a sustainable solution for water-scarce regions. Its high efficiency, low cost, and eco-friendly nature make it a promising candidate for addressing the global water crisis.

## Figures and Tables

**Table 1:** Evaporation efficiency of PS-PDA doped with different PDA concentrations.

Group	1-Top	1-Bottom	3-Top	3-Bottom	5-Top	5-Bottom	7-Top	7-Bottom
Efficiency	53%	53%	59%	54%	77%	78%	69%	63%

## Data Availability

Data are contained within the article.

## References

[1] Ahmed F.E., Hashaikeh R., Hilal N. (2019). Solar powered desalination—Technology, energy and future outlook. Desalination.

[2] Jiang Q.S., Derami H.G., Ghim D., Cao S.S., Jun Y.S., Singamaneni S. (2017). Polydopamine-filled bacterial nanocellulose as a biodegradable interfacial photothermal evaporator for highly efficient solar steam generation. J. Mater. Chem. A.

[3] Shannon M.A., Bohn P.W., Elimelech M., Georgiadis J.G., Marinas B.J., Mayes A.M. (2008). Science and technology for water purification in the coming decades. Nature.

[4] Elimelech M. (2006). The global challenge for adequate and safe water. J. Water Supply Res. Technol..

[5] Shang W., Deng T. (2016). Solar steam generation: Steam by thermal concentration. Nat. Energy.

[6] Ghasemi H., Ni G., Marconnet A.M., Loomis J., Yerci S., Miljkovic N., Chen G. (2014). Solar steam generation by heat localization. Nat. Commun..

[7] Li X., Xu W., Tang M., Zhou L., Zhu B., Zhu S., Zhu J. (2016). Graphene oxide-based efficient and scalable solar desalination under one sun with a confined 2D water path. Proc. Natl. Acad. Sci. USA.

[8] Ito Y., Tanabe Y., Han J., Fujita T., Tanigaki K., Chen M. (2015). Multifunctional Porous Graphene for High-Efficiency Steam Generation by Heat Localization. Adv. Mater..

[9] Zhang J.T., Qin Y.J., Pan W., Wang Z.B., Qi Y., He J.X., Zhang H.Q. (2023). Dual-Driven Functional Fabric with High Electrothermal and Photothermal Conversion Efficiency Modified by CuS Nanoparticles. ACS Appl. Polym. Mater..

[10] Simayee M., Zad A.I., Esfandiar A. (2023). Green synthesize of copper nanoparticles on the cotton fabric as a self-regenerating and high-efficient plasmonic solar evaporator. Sci. Rep..

[11] Simayee M., Zad A.I., Esfandiar A. (2023). Boosting-photothermal properties of Cu/Black TiO_2_ nanoparticles on biomimetics texture structure as high-performance and self-regenerating solar-evaporator. Sol. Energy.

[12] Shafaee M., Niazi Z., Asarnia M., Goharshadi E.K., Dehghani R. (2023). Modified pine cone with MnO_2_ nanoparticles as a photoabsorber for highly efficient seawater desalination and wastewater treatment. Appl. Phys. A.

[13] Mitra D., Chanda K., Bhattacharjee S., Bairi P., Chattopadhyay K.K., Chattopadhyay P. (2023). Enhanced interfacial evaporation and desalination by solar heat localisation using nitrogenated graphitic carbon and Co_3_O_4_ nanorods. Sol. Energy Mater. Sol. Cells.

[14] Jiang G., Fang X., Yu W., Xie H., Lei H. (2023). Magnetic recyclable Fe_3_O_4_@Ti_3_C_2_T_X_ nanoparticles for high-efficiency solar membrane distillation. Desalination.

[15] Joo B.S., Kim I.S., Han I.K., Ko H., Kang J.G., Kang G. (2022). Plasmonic silicon nanowires for enhanced heat localization and interfacial solar steam generation. Appl. Surf. Sci..

[16] Chen R., Wang X., Gan Q.M., Zhang T.Q., Zhu K.H., Ye M.M. (2019). A bifunctional MoS_2_-based solar evaporator for both efficient water evaporation and clean freshwater collection. J. Mater. Chem. A.

[17] Zhou L., Tan Y., Wang J., Xu W., Yuan Y., Cai W., Zhu S., Zhu J. (2016). 3D self-assembly of aluminium nanoparticles for plasmon-enhanced solar desalination. Nat. Photonics.

[18] Liu Y., Yu S., Feng R., Bernard A., Liu Y., Zhang Y., Duan H., Shang W., Tao P., Song C. (2015). A Bioinspired, Reusable, Paper-Based System for High-Performance Large-Scale Evaporation. Adv. Mater..

[19] Wang Z., Liu Y., Tao P., Shen Q., Yi N., Zhang F., Liu Q., Song C., Zhang D., Shang W. (2014). Bio-Inspired Evaporation Through Plasmonic Film of Nanoparticles at the Air-Water Interface. Small.

[20] Lead J.R., Batley G.E., Alvarez P.J.J., Croteau M.-N., Handy R.D., McLaughlin M.J., Judy J.D., Schirmer K. (2018). Nanomaterials in the environment: Behavior, fate, bioavailability, and effectsAn updated review. Environ. Toxicol. Chem..

[21] Selck H., Handy R.D., Fernandes T.F., Klaine S.J., Petersen E.J. (2016). Nanomaterials in the Aquatic Environment: A European Union-United States Perspective on the Status of Ecotoxicity Testing, Research Priorities, and Challenges Ahead. Environ. Toxicol. Chem..

[22] Ryu J.H., Lee Y., Kong W.H., Kim T.G., Park T.G., Lee H. (2011). Catechol-Functionalized Chitosan/Pluronic Hydrogels for Tissue Adhesives and Hemostatic Materials. Biomacromolecules.

[23] Li Y., Xu H., Li H., Zhong S. (2021). Controlled preparation and photothermal properties of polydopamine submicrospheres. Inorg. Chem. Commun..

[24] Liu Y., Ai K., Lu L. (2014). Polydopamine and Its Derivative Materials: Synthesis and Promising Applications in Energy, Environmental, and Biomedical Fields. Chem. Rev..

[25] Amin D.R., Higginson C.J., Korpusik A.B., Gonthier A.R., Messersmith P.B. (2018). Untemplated Resveratrol-Mediated Polydopamine Nanocapsule Formation. ACS Appl. Mater. Interfaces.

[26] Amin D.R., Sugnaux C., Lau K.H.A., Messersmith P.B. (2017). Size Control and Fluorescence Labeling of Polydopamine Melanin-Mimetic Nanoparticles for Intracellular Imaging. Biomimetics.

[27] Liu Y., Ai K., Liu J., Deng M., He Y., Lu L. (2013). Dopamine-Melanin Colloidal Nanospheres: An Efficient Near-Infrared Photothermal Therapeutic Agent for In Vivo Cancer Therapy. Adv. Mater..

[28] Ju K.-Y., Lee Y., Lee S., Park S.B., Lee J.-K. (2011). Bioinspired Polymerization of Dopamine to Generate Melanin-Like Nanoparticles Having an Excellent Free-Radical-Scavenging Property. Biomacromolecules.

[29] Yazdi M.K., Zare M., Khodadadi A., Seidi F., Sajadi S.M., Zarrintaj P., Arefi A., Saeb M.R., Mozafari M. (2022). Polydopamine Biomaterials for Skin Regeneration. ACS Biomater. Sci. Eng..

[30] Sun F., Lu J., Wang Y., Xiong J., Gao C., Xu J. (2021). Reductant-assisted polydopamine-modified membranes for efficient water purification. Front. Chem. Sci. Eng..

[31] Postma A., Yan Y., Wang Y., Zelikin A.N., Tjipto E., Caruso F. (2009). Self-Polymerization of Dopamine as a Versatile and Robust Technique to Prepare Polymer Capsules. Chem. Mater..

[32] Berthier E., Young E.W.K., Beebe D. (2012). Engineers are from PDMS-land, Biologists are from Polystyrenia. Lab Chip.

[33] An S., Lim J., Choi D., Hong H., Kim H.W., Park S.M., Rhie J.W., Kim D.S. (2017). Fabrication of polystyrene-based multi-well screening platform for micrometer-scale surface topographies promoting stem cell functions. Microelectron. Eng..

[34] Marquez C., Martin C., Linares N., De Vos D. (2023). Catalytic routes towards polystyrene recycling. Mater. Horiz..

[35] Ahmed D.S., El-Hiti G.A., Yousif E., Hameed A.S. (2017). Polyphosphates as Inhibitors for Poly(vinyl Chloride) Photodegradation. Molecules.

[36] Qiu J., Shi Y., Xia Y. (2021). Polydopamine Nanobottles with Photothermal Capability for Controlled Release and Related Applications. Adv. Mater..

[37] Zhu Y., Cheng Z., Weng W., Cheng K. (2018). A facile synthesis of polydopamine/TiO_2_ composite films for cell sheet harvest application. Colloids Surf. B.

[38] Wang R., Long Y.H., Zhu T., Guo J., Cai C., Zhao N., Xu J. (2017). Fabrication of oriented wrinkles on polydopamine/polystyrene bilayer films. J. Colloid Interface Sci..

[39] Moon S., Lee W., Ahn Y. (2016). Fabrication of Superhydrophobic Surface on Polydopamine-coated Al Plate by Using Modified SiO_2_ Nanoparticles/Polystyrene Nano-Composite Coating. Bull. Korean Chem. Soc..

[40] Li Z., Yang Y., Wang Z., Zhang X., Chen Q., Qian X., Liu N., Wei Y., Ji Y. (2017). Polydopamine nanoparticles doped in liquid crystal elastomers for producing dynamic 3D structures. J. Mater. Chem. A.

[41] Li Z. (2019). Polydopamine Based Photo-Responsive Shape Memory Polymer and the Achievement of Specific 3D Structures. Ph.D. Thesis.

[42] (2002). Salt Fusion: An Approach to Improve Pore Interconnectivity within Tissue Engineering Scaffolds. Tissue Eng..

[43] Xi Z.Y., Xu Y.Y., Zhu L.P., Wang Y., Zhu B.K. (2009). A facile method of surface modification for hydrophobic polymer membranes based on the adhesive behavior of poly(DOPA) and poly(dopamine). J. Membr. Sci..

[44] Chen Q.M., Pei Z.Q., Xu Y.S., Li Z., Yang Y., Wei Y., Ji Y. (2018). A durable monolithic polymer foam for efficient solar steam generation. Chem. Sci..

